# Multiple Mechanisms of the Synthesized Antimicrobial Peptide TS against Gram-Negative Bacteria for High Efficacy Antibacterial Action In Vivo

**DOI:** 10.3390/molecules26010060

**Published:** 2020-12-24

**Authors:** Rui Zhang, Xiaobo Fan, Xinglu Jiang, Mingyuan Zou, Han Xiao, Guoqiu Wu

**Affiliations:** 1 Medical School, Southeast University, Nanjing 210009, China; 220183546@seu.edu.cn (R.Z.); 101011951@seu.edu.cn (X.F.); 230189344@seu.edu.cn (X.J.); 230198759@seu.edu.cn (M.Z.); 230179771@seu.edu.cn (H.X.); 2 Jiangsu Provincial Key Laboratory of Critical Care Medicine, Southeast University, Nanjing 210009, China; 3 Department of Clinical Laboratory Medicine, Zhongda Hospital, Southeast University, Nanjing 210009, China

**Keywords:** antibacterial activity, membrane interaction, lipopolysaccharide, bacterial DNA

## Abstract

The emergence of drug-resistant bacteria emphasizes the urgent need for novel antibiotics. The antimicrobial peptide TS shows extensive antibacterial activity in vitro and in vivo, especially in gram-negative bacteria; however, its antibacterial mechanism is unclear. Here, we find that TS without hemolytic activity disrupts the integrity of the outer bacterial cell membrane by displacing divalent cations and competitively binding lipopolysaccharides. In addition, the antimicrobial peptide TS can inhibit and kill *E. coli* by disintegrating the bacteria from within by interacting with bacterial DNA. Thus, antimicrobial peptide TS’s multiple antibacterial mechanisms may not easily induce bacterial resistance, suggesting use as an antibacterial drug to be for combating bacterial infections in the future.

## 1. Introduction

Most antimicrobial drugs inhibit and finally kill bacteria by mainly interfering with specific intracellular substances in important biochemical cell processes [1]. However, bacteria can develop resistance simply by reducing or modifying antibiotic targets. For instance, beta-lactam antibiotics can bind to penicillin-binding proteins (PBPs), thus blocking the synthesis of cell wall mucin and resulting in the loss of the cell wall, expansion, and lysis of the bacteria, while gram-negative bacteria via porin mutations to resist antimicrobial drugs. Furthermore, using antibiotics widely in humans and the use of antibiotics without an appropriate treatment causes resistances to develop quickly [2,3]. In the last decade, pathogenic micro-organisms have exhibited dramatically increased resistances to most known antimicrobial drugs. In the era of drug resistance, the ability to control bacterial infections through antibiotics is weakened due to multidrug-resistant bacteria and the emergence of superbugs which could cause serious public health crises [4]. Due to the relatively slow development of new antibiotics, developing new, natural, and effective antibiotics with novel mechanisms is needed extremely urgently [5].

Antimicrobial peptides (AMPs), as an important effector molecule of innate immunity, are molecules with fewer than 50 amino acids and rapidly kill a broad range of bacteria, fungi, and enveloped viruses [6,7,8]. AMPs are considered a membrane-targeting reagent in most studies [9,10,11]. Generally, AMPs directly bind to anionic microbials on bacterial cell membranes via electrostatic interaction and then permeate the bacterial cell membrane to induce bacterial death. In addition, some cell-penetrating peptides show strong antibacterial activity across the cell membrane without disruption and intracellular interaction mechanisms [12,13,14,15]. It is difficult to develop bacterial resistance to these peptides because of their nonspecific sterilization mechanisms. They can be efficiently obtained by the natural world, so AMPs are one of the most prospective candidate drugs for the development of novel antibiotics [16]. Unfortunately, the high structural complexity, low antibacterial activity, and high hemolytic properties of natural AMPs hinder their development and clinical applications.

**Figure 1:**
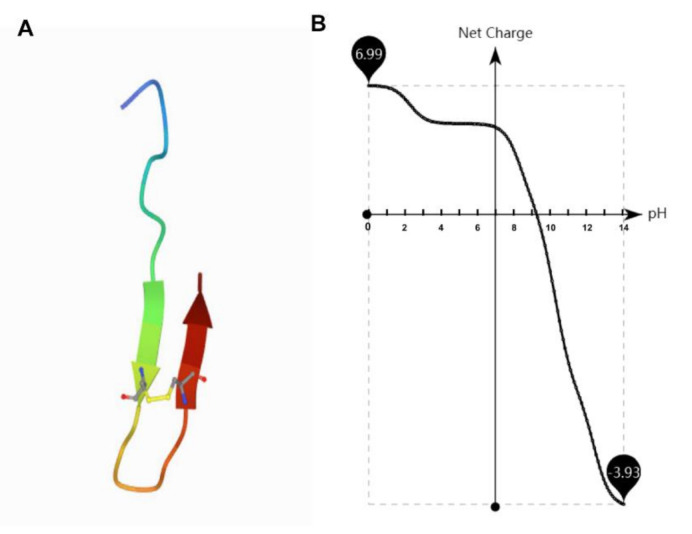
The structural and net charge of antimicrobial peptide TS. (**A**) Structural assembly simulations of antimicrobial peptide TS. (**B**) The net charge of antimicrobial peptide TS is related to the pH.

Antimicrobial peptide TS (GSKKPVPIIYCNRRSGKCQRM) is synthesized by substituting an amino acid for thanatin (GSKKPVPIIYCNRRTGKCQRM), which is isolated from the hemipteran insect *Podisus maculiventris* [17]. TS was found with an improved antimicrobial activity and its antimicrobial activity was less susceptible to cations and pH [18,19]. In this study, we find that TS features a β-helix structure constrained by disulfide bonds between Cys11 and Cys18 (Figure 1A) through data simulation (https://zhanglab.ccmb.med. umich.edu/I-TASSER/). Although the structure of TS is relatively simple, it has high antibacterial activity, low toxicity as well as inhibited LPS activity [20]. Up to now, its antibacterial mechanism has been unclear. In this study, the physicochemical properties of TS are elucidated, along with the localization of TS on bacterial cells, the interaction between TS and bacterial cell walls (especially LPS), the binding ability between DNA and TS, and the antibacterial effect in vivo. We find that antimicrobial peptide TS has stable physicochemical properties and plays an efficient bactericidal role through multiple mechanisms of action, which is important for facilitating the exploitation and use of new peptides for the treatment of clinical microbial infections and supporting the development of new antibiotics.

## 2. Results and Discussion

### 2.1. Antibacterial and Bactericidal Activity

**Figure 2:**
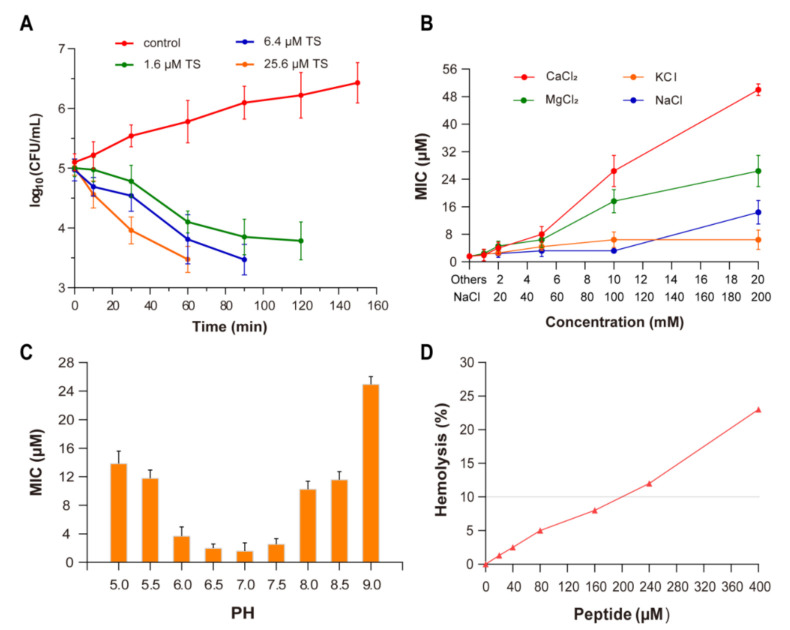
Antibacterial activity and physicochemical properties of antimicrobial peptide TS. (**A**) Time killing curve for *E. coli* ATCC 25922 in a MH broth with antimicrobial peptide TS (control, no antibiotic). (**B**) The effect of the ions Ca^2+^, Mg^2+^, K^+^, and Na^+^, on the antimicrobial activity of TS against *E. coli* ATCC 25922. (**C**) The effect of pH on the antimicrobial activity of TS against *E. coli* ATCC 25922. (**D**) The hemolytic activity of antimicrobial peptide TS.

As predicted, antimicrobial peptide TS exhibits significant antimicrobial activity against gram-negative bacteria, including the *E. coli* ATCC 25922 and K. pneumoniae ATCC 700603 strains at 1.6 μM for both for the minimum inhibitory concentration (MIC) values (Table 1), whereas *S. aureus* strains show no obvious susceptibilities to it. The time killing curve of *E. coli* ATCC 25922 in a Mueller–Hinton (MH) broth with antimicrobial peptide TS showed rapid bacterial action (Figure 2A). At the MIC level, TS could kill almost all bacteria within 120 min. In addition, at MIC values of 1.6 and 6.4 μM TS even exhibited rapid bacterial reduction from 10^5^ to 10^4^ colony forming units (CFU)/mL at 60 min and 30 min, respectively, which means that the bacterial death rate was more than 99%. Thus, for 25.6 μM, 6.4 μM, and even 1.6 μM, antimicrobial peptide TS has obvious and rapid inhibitory and bactericidal effects on the growth of *E. coli* ATCC 25922.

### 2.2. Effect of Ions

In the absence of cations and without pH change, the MIC value of antimicrobial peptide TS was 1.6 μM. With the increase in the cation concentration, the antimicrobial activity of TS against *E. coli* ATCC 25922 decreased (Figure 2B). The normal growth of bacteria is not affected by these cations. The activity of TS was not significantly interfered with in the presence of 10 mM of potassium ions (K^+^) or 200 mM of sodium ions (Na^+^). When the divalent cation concentration was less than 5 mM, there was almost no inhibitory effect on antimicrobial peptide TS; however, the MIC of TS rapidly increased when increasing the calcium ion (Ca^2+^) and magnesium ion (Mg^2+^) concentrations, presenting an inhibition effect for TS against *E. coli* ATCC 25922.

Since NaCl and KCl are the predominant salts and Ca^2+^ and Mg^2+^ are major divalent cations in vivo, an antimicrobial peptide’s ability to tolerate salt should not be neglected. Cationic antimicrobial peptides such as α-defensin HD-5 and cathelicidin LL-37 are salt sensitive and would lose their antibacterial activity in high salt concentrations [21,22]. In this study, we find that antimicrobial peptide TS is highly active in such a physiological environment. It is worth noting that the antagonistic effects of Ca^2+^ with the increase of in concentration had an obvious effect on the antimicrobial activities of TS, and the mechanism of this effect needs further research.

### 2.3. Effect of pH

At pH values from 6.0 to 7.5, the MIC values of antimicrobial peptide TS did not significantly fluctuate (Figure 2C). The antimicrobial activity of TS was affected in strongly alkaline and acidic conditions. Antimicrobial peptide TS features a pH-dependent activity. The growth of bacteria in the positive control wells shows that there was no change in impact due to pH. Similar effects from pH changes on cationic peptide activity have been reported by other researchers [23,24]. Surprisingly, TS activity was substantially greater at pH 6.0 than pH 8.0, which shows that the antimicrobial activity of TS was a little higher under acidic conditions than basic conditions, although it maintained good antibacterial activity under a wide range of pH levels. Cationic antimicrobial peptides exhibit antimicrobial activity through electrostatic interaction with bacterial cell membranes [12,13,14,15], while the net charge of antimicrobial peptide TS decreased gradually as the pH increased (Figure 1B), which explains why the antimicrobial activity decreases with the gradual alkalinity of the pH. This also explains TS peptides are synthesized with high isoelectric point (IP) charges (Table 2).

### 2.4. Hemolytic Activity

As an examination of toxicity, the hemolytic activity of antimicrobial peptide TS was investigated using human red blood cells (RBCs) (Figure 2D). Antimicrobial peptide TS was well above the MIC (160 μM), showing no significant hemolytic activity (<10%) against human blood cells, which have low cytotoxicity and might be applicable as an antimicrobial therapeutic drug.

### 2.5. Localization of Antimicrobial Peptide TS on Bacterial Cells

**Figure 3:**
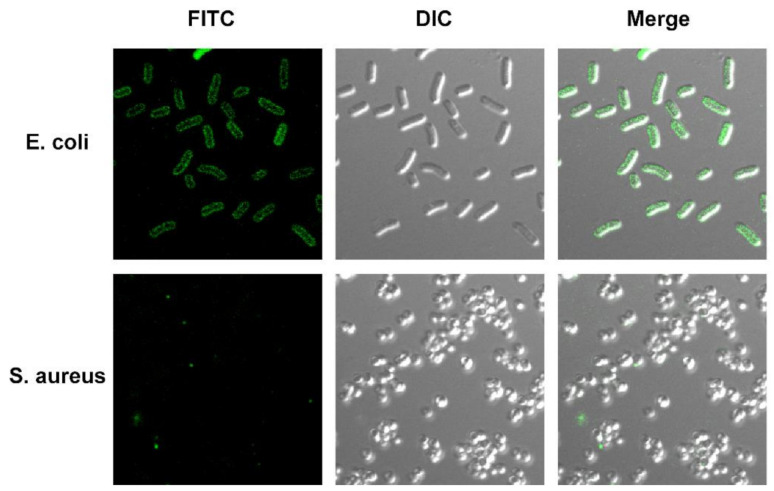
Fluorescence confocal laser scanning microscope of FITC-labeled antimicrobial peptide TS in *E. coli* cells for localization. *E. coli* ATCC 25922 and S. aureus ATCC 29213 treated with FITC-labeled antimicrobial peptide TS for 10 min.

To observe the localization of the action site of antimicrobial peptide TS in *E. coli* more accurately, a green fluorescent probe fluorescein isothiocyanate (FITC) was labeled to the N-terminal site of antimicrobial peptide TS to detect its action site via confocal laser scanning microscopy. The MIC of this derivative against *E. coli* was 1.6 μM, which maintained a good antibacterial activity, so the large intestine was selected for fluorescence microscope detection. The staining pattern (Figure 3) revealed that the FITC fluorescence was concentrated on *E. coli* ATCC 25922 bacterial cell surfaces, and the staining time was very fast, which was only 5 min. The rapid phenomenon of dyeing is consistent with the rapid effectiveness of sterilization. Furthermore, *S. aureus* ATCC 29213 (Figure 3) was used as a negative control since antimicrobial peptide TS is not effective against it (Table 1), and it was stained without any fluorescence.

It is noteworthy that FITC, as an effective probe, is unable to penetrate a bacterial cytoplasmic membrane unless the cell membrane is disrupted [25,26]. The image suggests that antimicrobial peptide TS can specifically bind to substances on the surfaces of bacteria. It is noteworthy that FITC could not affect the antibacterial and bactericidal activities of antimicrobial peptide TS (data not shown). Overall, our results show that the bacterial cell membrane may be the main action site of antimicrobial peptide TS, which can bind to bacterial cell membranes and accumulate there.

### 2.6. Morphological Changes of Antimicrobial Peptide TS on Bacterial Cell Membrane

**Figure 4:**
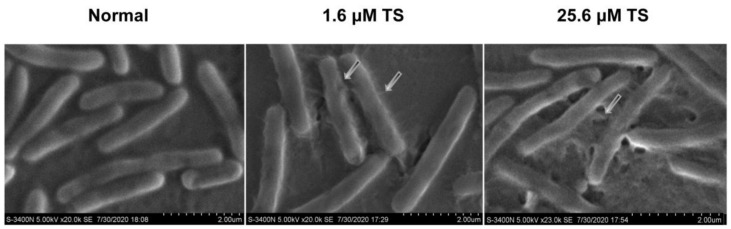
Scanning electron microscopy of *E. coli* ATCC 25922 and *E. coli* ATCC 25922 treated with 1.6 μM or 25.6 μM of antimicrobial peptide TS.

To further inspect the effect of peptides on the destruction of bacterial cell membranes, SEM was used to allow visualization the morphological changes which appear in the bacteria after treatment with antimicrobial peptide TS (Figure 4). In the absence of peptide, *E. coli* ATCC 25922 cell surfaces were normal, smooth, uniform, complete, and full; however, the notable features of TS-treated *E. coli* ATCC 25922 were significant, including rough, distorted, and deformed characteristics that was not seen in the control. Large filament amounts could be seen on the surface of *E. coli* ATCC 25922 treated by 1.6 μM TS, and the filaments may be the aggregation of TS on the cell surface, which is consistent with the confocal laser scanning microscopy results. We could even identify the rupturing of some bacterial cell membranes. The protoplasm of the bacteria treated with 25.6 μM TS is obviously sticky and cell aggregation and adhesion occurred.

These results further reveal that bacteria treated with antimicrobial peptide TS were killed by the destruction of *E. coli* cell membrane surface, which caused the decomposition of the bacteria itself. It is well known that gram-negative bacteria have both an outer membrane (OM) and inner membrane (IM), and the specific effects of antimicrobial peptides on the IM and OM deserve further attention and discussion.

### 2.7. Permeabilization of the Outer Membrane (OM)

**Figure 5:**
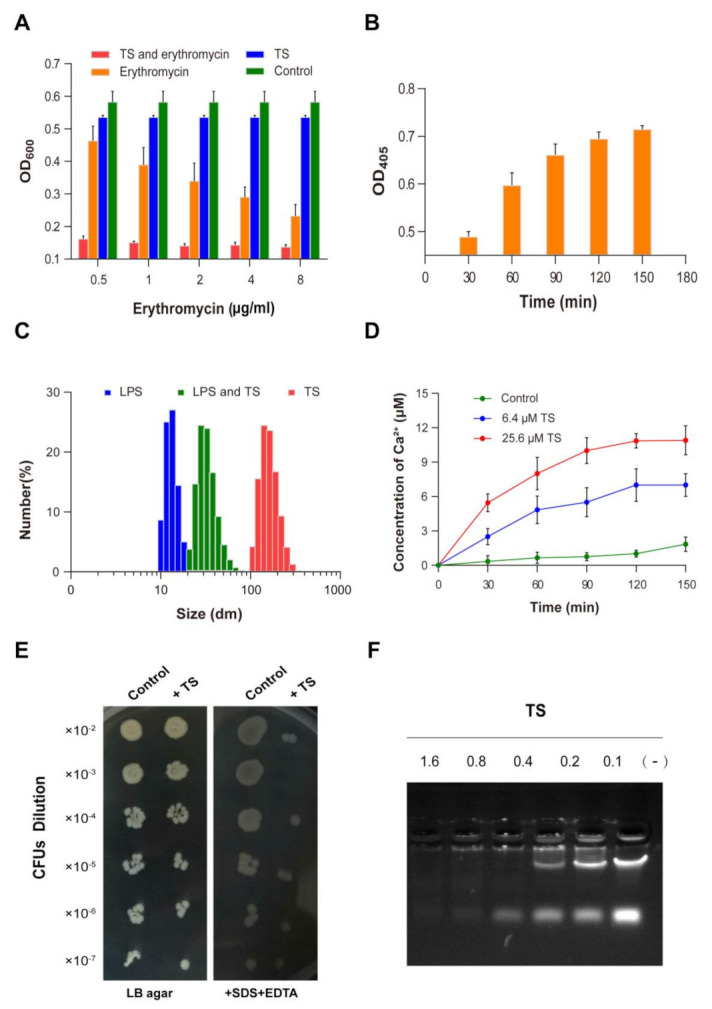
Antibacterial mechanism of antimicrobial peptide TS. (**A**) The effect of TS on permeabilizing the outer membrane of *E. coli* ATCC 25922. (**B**) The effect of TS on permeabilizing the inner membrane of *E. coli* ATCC 25922. (**C**) Interaction of antimicrobial peptide TS with lipopolysaccharides (LPSs). (**D**) Release of Ca^2+^ and K^+^ from TS-treated *E. coli* ATCC 25922. (**E**) The sensitivity of TS-treated *E. coli* ATCC 25922 to SDS/EDTA. (**F**) Gel retardation analysis for the binding of TS to the DNA of *E. coli* ATCC 25922.

The effect of antimicrobial peptide TS against the OM of *E. coli* ATCC 25922 was investigated using an erythromycin antibiotic probe. Erythromycin has a weak ability to completely penetrate the enterobacterial OM, but it is easily traverses a damaged bacterial cell outer membrane [27]. At approximately 0.5 for the MIC of TS, the obvious inhibitory effect could be hardly observed in the growth of the bacteria, while erythromycin plays an antibacterial role in a dose-dependent manner (Figure 5A). Comparatively, the growth of *E. coli* ATCC 25922 was inhibited significantly with the addition of TS and erythromycin, with only a few bacterial growths. In particular, the inhibitory effect of antimicrobial peptide TS was enhanced by over four times in the presence of erythromycin.

Our results show that antimicrobial peptide TS was effective at permeabilizing the OM of *E. coli*, which promotes higher erythromycin absorption and use, and improves the antimicrobial activity of erythromycin, providing guidance for the combined application of erythromycin and the peptide.

### 2.8. Permeabilization of the Inner Membrane (IM)

The results regarding the bacterial IM integrity were examined and are given in Figure 5B. It was found that antimicrobial peptide TS induced an increase in extracellular B-galactosidase in a time-dependent manner. At 30 min, extracellular β-galactosidase could be detected after treatment with antimicrobial peptide TS. There was a rapid release of B-galactosidase by less than 60 min. At about 150 min, levels of β-galactosidase in the extracellular compartment reached a steady state. At the same time, there was little change in the optical density (OD) value for the control group, which means that normal bacteria do not release β-galactosidase (not shown).

One of the key steps to achieve membrane permeability is for peptides to enter and damage the bacterial IM. Generally, the cytoplasmic β-galactosidase does not pass through the intact endometrium of *E. coli*, which can be tested extracellularly when the IM of the bacteria is destroyed. 2-Nitrophenyl β-d-galactopyranoside (ONPG) can be hydrolyzed to o-nitrophenol by the release of β-galactosidase, and its color change can be determined by spectrophotometry. Our results confirm that antimicrobial peptide TS can permeabilize the IM of *E. coli.* Additionally, the inner membrane of bacteria could be permeabilized by other antimicrobial peptides, such as chensinin-1b and buforin 2 [9,11].

### 2.9. Interaction of Peptides with Lipopolysaccharides

To study the effect of antimicrobial peptides on the structure of LPSs, a molecular particle size measuring instrument was used to observe the depolymerization of antimicrobial peptides on LPS micelles. The overnight placement of LPSs allowed LPS monomers to form molecular aggregates. LPS initially existed in one primary size with an average size of 17 nm, while antimicrobial peptide TS agglomerated into particles of about 200 nm in size (Figure 5C). The addition of TS changed the LPSs into bigger aggregates, with 25 nm comprising the most abundant particles.

The binding and permeation of antimicrobial peptide TS with the bacterial membrane is the first link of the interaction of antimicrobial peptides and bacteria [28], and it is also the key step of the antibacterial mechanism [29]. Lipopolysaccharides, a major constituent of OM of gram-negative bacteria, are considered to be the first permeability barrier that blocks the entry of harmful reagents including antibiotics and host proteins [30,31]. Majority cationic antibacterial peptides can bind to negatively charged LPSs on the OM of gram-negative bacteria [32,33,34]. It is worth noting that antimicrobial peptide TS can neutralize LPSs to move through the OM LPS barrier to the bacterial intima and then destroy the intima to kill the bacteria. In addition, several scientists have found that the positively charged biological amino acids of AMPs bind to negatively charged LPSs on the OM of gram-negative bacteria through Mg^2+^ and Ca^2+^ ion competition [34,35]. The same result can be observed in this experiment (Figure 5D). The release of bacterial calcium ions induced by TS occurred in a time- and concentration-dependent manner. In addition, the previous experiments showed that the increased concentration of Ca^2+^ and Mg^2+^ ions had a negative effect on TS antibacterial activity, which was also confirmed by deduction via the antimicrobial peptide TS being bound to LPS by means of ion competition.

### 2.10. Susceptibility to Detergents

The antimicrobial peptide TS-treated cells barely grew on the Luria–Bertani (LB) agar plate containing SDS/EDTA compared with untreated cells (Figure 5E). The TS-treated cells showed significantly higher sensitivity to SDS/EDTA, which is consistent with an impaired OM permeability barrier and improved lethal effects of IM exposure to a detergent. The cells also showed increasing sensitivity to erythromycin (Figure 5A). This further elucidates the properties of antimicrobial peptide TS in combination with other antibiotics to enhance antimicrobial activity.

### 2.11. Interaction of Antimicrobial Peptide TS with Cellular DNA

Positively, it was reported that charged antimicrobial peptides can directly bind to bacterial DNA, thus inhibiting cellular biopolymer synthesis and functioning [12,13]. To assess the binding ability of antimicrobial peptide TS to the DNA of *E. coli*, increasing concentrations of antimicrobial peptide TS were incubated with bacterial DNA. At several weight ratios (antimicrobial peptide TS/DNA) up to 1.6:0.5, the electrophoretic mobility of bacterial DNA was slightly inhibited, and the migration of bacterial DNA was hardly observed in the gel (Figure 5F). When the weight ratio was 0.8:0.5, only a small part of the bacterial DNA migrated into the gel. When the ratios were 0.4:0.5, 0.2:0.5, and 0.1:0.5, the DNA could migrate to the gel and the DNA migration increased as the peptide decreased, suggesting that antimicrobial peptide TS binds with DNA in a concentration-dependent manner. These results were similar to those of the previously reported antimicrobial peptide LBP/BPI [15]. In other studies, a cathelicidin-derived peptide has been shown to be effective against penicillin-resistant bacteria through DNA binding and decrease the replication of a plasmid containing an antimicrobial resistance gene [36].

### 2.12. Antimicrobial Peptide TS Protects Mice Infected with E. coli ATCC 25922

**Figure 6:**
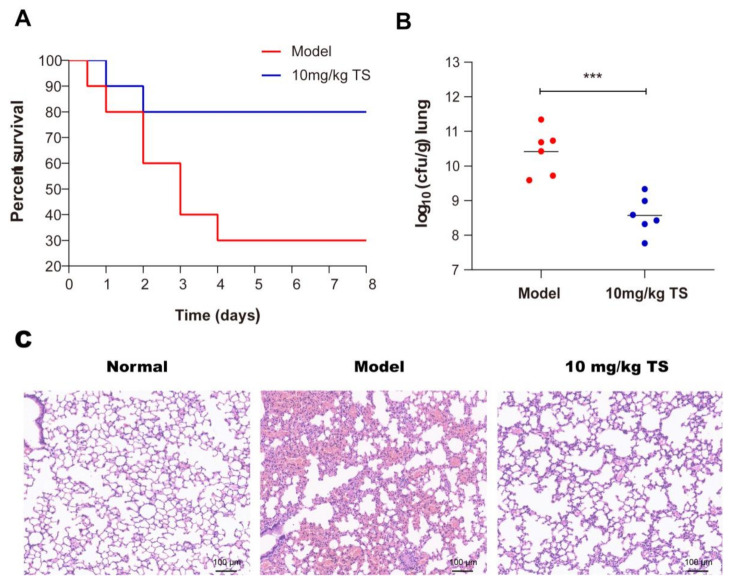
Antimicrobial peptide TS protects mice infected with *E. coli* ATCC 25922. (**A**) Survival rates for the *E. coli* ATCC 25922 pneumonia model. (**B**) Bacterial loads of the lungs of TS-treated *E. coli* ATCC 25922-infected mice were examined at 24 h after infection. (**C**) H&E staining was used to detect lung morphologies of the *E. coli* ATCC 25922 pneumonia model after TS treatment. *** *p* < 0.001.

We tested the in vivo therapeutic effect of antimicrobial peptide TS in an *E. coli* ATCC 25922 pneumonia model. The survival rate increased from 30% to 80% after treatment with 10 mg/kg of TS in *E. coli* ATCC 25922-infected mice (Figure 6A). Lung samples were collected 24 h after exposure to examine bacterial CFUs after treatment, and the results show that the titers of bacteria in the lungs of mice were significantly reduced after treatment (Figure 6B). Histological staining indicated the therapeutic outcome (Figure 6C). In the model group, significant pathological changes were observed, including mass inflammatory cell infiltration, alveolar fusion, dilation, and congestion. By contrast, the TS therapy rescued the pathological injury. The increasing survival rates with TS therapy were associated with decreased bacterial titers and limited pneumonia exacerbations in infected mice lungs.

Despite considerable efforts by healthcare authorities to implement prevention guidelines, ventilator-associated pneumonia (VAP) has always been the most frequent life-threatening nosocomial infection [37]. More than 60% of hospital-acquired pneumonia infections are caused by Gram-negative bacteria [38]. In the past, the most common gram-negative bacteria causing VAP was *Pseudomonas aeruginosa*; however, *Enterobacteriaceae*, including *E. coli*, are increasingly involved in VAP [39,40]. The antimicrobial peptide TS could treat the bacterial pneumonia caused by *E. coli* well and provide a new option for clinical treatment in the future.

## 3. Materials and Methods

### 3.1. Synthesis and Purification of Antimicrobial Peptide TS

Antimicrobial peptide TS (GSKKPVPIIYCNRRSGKCQRM) was synthesized using standard Fmoc solid-phase peptide synthesis protocols (Science Peptide Co. Ltd., Shanghai, China), and purity was assayed by HPLC and mass spectrometry. After freeze-drying, the purity degree was over 98%. Then, TS was suspended in sterile distilled water and stored at −20 °C.

### 3.2. Bacterial Strains

*E. coli* ATCC 25922, *K. pneumonia* ATCC 700603, *P. mirabilis* ATCC 12453, *P. aeruginosa* ATCC 27853, and *S. aureus* ATCC 29213 were used throughout this experiment. They were obtained from the Institute of Microbiology, China Pharmaceutical University. 

### 3.3. Minimum Inhibitory Concentration (MIC)

The MIC values of TS were identified through a standard micro-dilution method in sterilized 96-well plates according to the broth micro-dilution method of the Clinical and Laboratory Standards Institute [41]. An exponential phase of growth bacteria sample was diluted with a fresh MH broth to a final concentration of 5 × 10^5^ CFU/mL. After 16 h of incubation at 37 °C under different concentrations of antimicrobial peptide TS, MICs were defined as the lowest antimicrobial peptide concentration that resulted in no visible bacteria growth.

### 3.4. Time Killing Curve Assay

The exponential phase *E. coli* ATCC 25922 was diluted with a fresh MH broth to approximately 10^5^ CFU/mL. Various concentrations of antimicrobial peptide TS were added to the dilution of bacterial fluid and the final concentrations (1.6 μM, 6.4 μM and 25.6 μM) for 0, 10, 30, 60, 90, 120, and 150 min in a microplate format. Antimicrobial peptide TS was untreated as a negative control. The culture was appropriately diluted and the CFU number was measured by the colony count on the plate.

### 3.5. Effect of Ions and pH

The effects of metal ions and pH on the activity of antimicrobial peptides were tested by measuring the MICs at different salt concentrations and pH values. The MH broth was altered by adding salt (CaCl_2_, MgCl_2_, KCl, NaCl), HCl, or NaOH. CaCl_2_, MgCl_2_, and KCl were added in concentrations of 0, 1, 2, 5, 10, and 20 mM. The concentrations of NaCl were 0, 10, 20, 50, 100, and 200 mM. Concentrations were detected at pH 5–8.

### 3.6. Hemolysis Assay

The hemolytic activity of antimicrobial peptide TS was determined using human erythrocytes. The erythrocytes were obtained by centrifuging blood and washing three times with phosphate buffer salt (PBS), and then incubating with diluted antimicrobial peptide TS in PBS for 60 min at 37 °C. The negative control was an erythrocyte suspension incubated in PBS, and the positive control was incubated in 1% Triton X-100 and was a standard for 100% hemolysis. Hemolysis was evaluated by measuring the optical density of the erythrocyte supernatant at 405 nm after centrifugation. The percentage of hemolysis (%) = (OD405 in peptide-OD405 in PBS)/(OD405 1% Triton X-100-OD405 in PBS) × 100%.

### 3.7. Confocal Laser Scanning Microscopy

Antimicrobial peptide TS was labeled with FITC, which was a step performed by the source company (Science Peptide Co. Ltd., Shanghai, China). The exponential phase *E. coli* ATCC 25922 and *S. aureus* ATCC 29213 (10^8^ CFU/mL) were centrifugated and washed with PBS three times, respectively, and then incubated with FITC-labeled antimicrobial peptide TS (concentration of 16 μg/mL) in the dark for 10 min. After washing with PBS three times, the samples could be observed by laser confocal microscope.

### 3.8. Scanning Electron Microscopy (SEM)

*E. coli* ATCC 25922 culture mediums were diluted with PBS to 10^8^ CFU/mL and then incubated with different concentrations of TS (1.6 μM, 25.6 μM) at 37 °C at 200 rpm for 60 min. The bacteria were treated without TS as a negative control. Following that, the bacteria were harvested after centrifugation at 5000 rpm for 5 min and being washed three times with PBS. Then, the bacteria were fixed with 5% glutaraldehyde for 3 h at 4 °C, and ethanol was added to dehydrate the samples. The morphological changes of the bacteria were observed under a scanning electron microscope (Hitachi S-3400N, Tokyo, Japan).

### 3.9. OM Permeabilization Assay

Erythromycin, as an antibiotic probe, was used to study whether antimicrobial peptide TS was effective against the OMs of bacteria. This experiment was measured with four experimental groups. Antimicrobial peptide TS and erythromycin were first dissolved in sterile water for use. In the first group, antimicrobial peptide TS (the final concentration 0.8 μM) and erythromycin with different concentrations (final concentrations of 0.5 mg/mL, 1 mg/mL, 2 mg/mL, 4 mg/mL, and 8 mg/mL, respectively) were added into the bacterial culture media, which were overnight cultures of *E. coli* ATCC 25922 that were diluted with a LB to 10^8^ CFU/mL. In the second group, different concentrations of erythromycin (same concentration as the first group) were added. In the third group, antimicrobial peptide TS (final concentration of 0.8 μM) was added. The fourth group was combined with the same volume of sterile water without antimicrobial peptide TS and erythromycin. Then, the four groups were incubated at 37 °C for 10 h and the bacterial growth was detected at 630 nm by a microplate reader.

### 3.10. IM Permeabilization Assay

Using ONPG as a substrate, the release of B-galactosidase from *E. coli* in the culture medium was measured to determine the bacterial endometrial permeability [42]. The exponential phase *E. coli* ATCC 25922 (10^8^ CFU/mL) that was incubated overnight was centrifugated and then washed with PBS three times, and the harvested bacteria were resuspended with PBS to 10^10^ CFU/mL. In addition, ONPG (1.5 mM) and antimicrobial peptide TS (6.4 μM) were added to the bacterial suspensions at 37 °C for 10, 30, 60, 90, 120, and 150 min. The bacteria without antimicrobial peptide TS were used as a negative control. After sampling and centrifuging at different reaction time points, OD405 was measured using a microplate reader to evaluate the production of o-nitrophenol.

### 3.11. DLS

The interaction of TS with lipopolysaccharides (LPS) from *E. coli* serotype O111:B4 was assessed by DLS measurements with a Zetasizer instrument (DT310, San Francisco, SF, USA). First, LPSs were extensively solubilized in PBS (pH 6.0). A final concentration of 0.5 μM was sonicated at 60 °C for 40 min. After 3–4 temperature cycles between 60 °C and 24 °C (room temperature), the lipid samples were stored in 4 °C for at least 12 h. A final concentration of 2 μM antimicrobial peptide TS was dissolved in PBS (pH 6.0). Finally, the antimicrobial peptide and LPS were incubated for 30 min. DLS experiments were used to detect changes in the particle sizes of the peptides and LPSs.

### 3.12. Calcium Ion Release Assay

The exponential phase *E. coli* ATCC 25922 (10^10^ CFU/mL) was centrifugated, washed three times, and then resuspended in 0.9% sterile saline. The bacterial suspension and antimicrobial peptide TS (final concentration: 6.4 μM, 25.6 μM) were incubated at 37 °C for 30, 60, 90, 120, and 150 min, respectively. Appropriate amounts of the bacterial suspensions were taken at multiple time points and centrifugated at 3000 rpm for 10 min, and then the supernatants were determined by atomic absorption spectrometry.

### 3.13. Susceptibility to Detergents

*E. coli* ATCC 25922 was grown in a LB broth at 37 °C for 10 h, adjusted to 10^7^ CFU/mL. Diluent and antimicrobial peptide TS (6.4 μM) were incubated for 1 h and coated onto LB medium plate or modified LB medium plate that contained 0.5 mM EDTA and 0.5% SDS through increasing dilutions. Untreated bacteria were used as negative controls.

### 3.14. DNA Binding Assay

A gel retardation experiment was used to evaluate the peptide’s ability to bind with intracellular DNA [14]. In brief, varying amounts of peptides (1.6, 0.8, 0.4, 0.2, 0.1, 0 μg/mL) were mixed with a certain concentrations of *E. coli* ATCC 25922 DNA (0.5 μg/mL) at a volume ratio of 1:1, incubated at 37 °C for 30 min, and then separated by 1.0% agarose gel electrophoresis including ethidium bromide (EtBr: 0.5 μg/mL) in a TAE (Tris- acetic acid-EDTA) buffer. Wells containing only bacterial DNA were used as negative controls.

### 3.15. Animal

Adult female ICR (Institute of Cancer Research) mice weighing 20–25 g were obtained from Qinglong Mountain Animal Farm (Nanjing, China). All animals were caged in groups at random, acclimatized for 1 week in an animal environmental control unit (temperature of 23 ± 1 °C; relative humidity of 50 ± 10%; light–dark cycle of 12 h) and had access to water and food ad libitum throughout the study.

### 3.16. Mouse Pneumonia Model

The mice were anaesthetized via an intraperitoneal injection with 10 mg/mL pentobarbital (40 mg/kg) and percutaneous lung punctures were infected with 1 × 10^8^ CFU/mL *E. coli* ATCC 25922 in 30 μL of MH. After bacterial exposure, antimicrobial peptide TS was intraperitoneally administered at 10 mg/kg in sterile water. Control mice were injected with the same volume of sterile water but without peptides. Mice were monitored for their lung bacterial numbers at 24 h after bacterial post exposure. Six mice in each group were sacrificed, and the lungs were immediately removed and homogenized. The samples were spread onto plates in fixed volumes with serial dilutions. The bacterial numbers were counted for CFU/mL determination. The lungs were fixed in 10% neutral buffered formalin, embedded in paraffin wax, and processed for hematoxylin and eosin (HE) staining. The survival of the remaining 10 mice in each group for 7 days was observed.

### 3.17. Statistical Analysis

Each experiment was repeated in triplicate. All data were analyzed by using the SPSS 20.0 statistical software IBM, Armonk, NY, USA) Results are expressed with the standard deviation (SD) and *p* < 0.05 was considered statistically significant.

## 4. Conclusions

**Figure 7:**
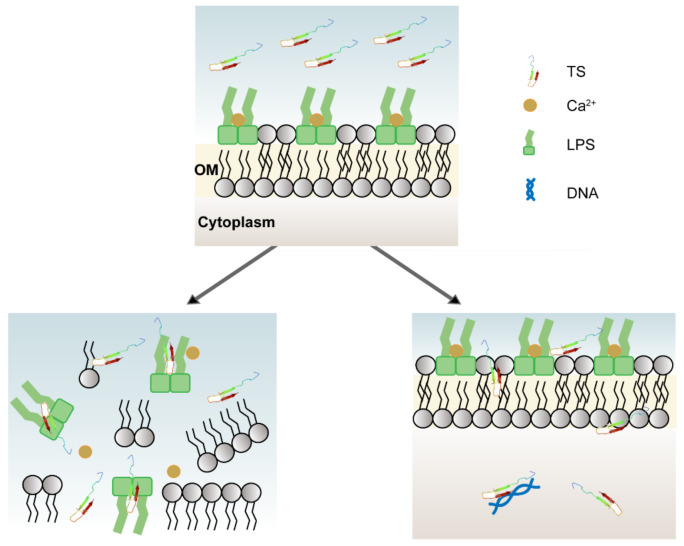
Multiple mechanisms of antimicrobial peptide TS. TS can inhibit and kill bacteria by displacing divalent cations, competitively binding lipopolysaccharides, and from within by interacting with bacterial DNA.

The antimicrobial and hemolytic results obtained here demonstrate that the antibacterial peptide TS interacts with bacterial outer membranes (even with LPSs) and inner membranes, causing damage to the cell membranes and promoting the release of content to achieve a bactericidal process without conferring an obvious hemolytic effect (Figure 7). Moreover, the antimicrobial peptide TS may bind to DNA, exerting the role of normal physiological anabolism and inducing metabolic disorder for bacteria. Antimicrobial peptide TS was used to treat lung infections and increased survival for pneumonia-infected mice. Further studies are needed to confirm the effects of VAP components and membrane proteins on the antibacterial mechanism. Further study is required to better understand the antibacterial activity mechanisms presented here. These results indicate that the antimicrobial peptide TS has multiple targets against *E. coli* ATCC 25922 and is a multi-functional antibacterial pathogen inhibitor. Compared with single antibiotics, it is not easy for bacteria to develop resistance, so TS has broad application prospects in the medical field.

## Figures and Tables

**Table 1:** Minimum inhibitory concentration (MIC) values of TS and other antibiotics.

Bacteria	MIC				
	TS (μg/mL)/(μM)	Ampicillin (μg/mL)	Erythromycin (μg/mL)	TrimeThoprim (μg/mL)	AzithromyCin (μg/mL)
*E. coli* (ATCC 25922)	4/1.6	8	>64	<0.5	32
*K. pneumonia* (ATCC 13883)	4/1.6	8	>64	15.6	32
*P. aeruginosa* (ATCC 27853)	32/12.8	8	>64	8	>64
*E. faecium* (ATCC 29212)	64.0/25.6	2	4	2	8
*S. aureus* (ATCC 29213)	>64	2	1	2	1

**Table 2:** Basic properties of antimicrobial peptide TS.

	Sequence	Molecular Weight	Extinction Coefficient	GRAVY	Isoelectric Point
**TS**	GSKKPVPIIYCNRRSGKCQRM	2419.88	1400 M^−1^cm^−1^	−0.9	10.87

## Data Availability

The analyzed data sets generated during the study are available from the corresponding author on reasonable request.

## Sample Availability

Samples of the compounds are available from the authors.
